# Where and why are species' range shifts hampered by unsuitable landscapes?

**DOI:** 10.1111/gcb.16220

**Published:** 2022-05-19

**Authors:** Jenny A. Hodgson, Zoë Randle, Chris R. Shortall, Tom H. Oliver

**Affiliations:** ^1^ Department of Evolution, Ecology and Behaviour University of Liverpool Liverpool UK; ^2^ Butterfly Conservation Wareham UK; ^3^ Rothamsted Research Harpenden UK; ^4^ School of Biological Sciences University of Reading Reading UK

**Keywords:** barrier, climate change, conductance, connectivity, dispersal, habitat, land use, lepidoptera, moth, permeability, range shift

## Abstract

There is widespread concern that species will fail to track climate change if habitat is too scarce or insufficiently connected. Targeted restoration has been advocated to help species adapt, and a “conductance” metric has been proposed, based on simulation studies, to predict effective habitat configurations. However, until now there is very little empirical evidence on how the configuration of habitat is affecting expansion at species' cool range margins. We analysed the colonisation events that have occurred in continuously monitored trap locations for 54 species of southerly distributed moths in Britain between 1985 and 2011. We tested whether the time until colonisation was affected by attributes of each species, and of intervening landcover and climate between the trap and the baseline distribution (1965–1985). For woodland species, the time until colonisation of new locations was predicted by the “conductance” of woodland habitat, and this relationship was general, regardless of species' exact dispersal distances and habitat needs. This shows that contemporary range shifts are being influenced by habitat configuration as well as simple habitat extent. For species associated with farmland or suburban habitats, colonisation was significantly slower through landscapes with a high variance in elevation and/or temperature. Therefore, it is not safe to assume that such relatively tolerant species face no geographical barriers to range expansion. We thus elucidate how species' attributes interact with landscape characteristics to create highly heterogeneous patterns of shifting at cool range margins. Conductance, and other predictors of range shifts, can provide a foundation for developing coherent conservation strategies to manage range shifts for entire communities.

## Graphical abstract

We show how moth species’ attributes interact with landscape characteristics ‐ landcover patterns and temperature ‐ to facilitate or hamper range expansion at cool range margins in the UK. We dissect how the colonisation of new locations differs between species and between landscapes: there is considerable variation even in this relatively mobile taxonomic group. For woodland species, the time until colonisation of new locations was predicted by the ‘conductance’ of the woodland habitat network. Therefore the conductance metric offers a way to evaluate landscapes and improve their functioning under climate change.

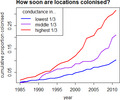

see also the Commentary: https://doi.org/10.1111/gcb.16180.

## 1 INTRODUCTION

Many species' ranges have shifted poleward in recent decades as the climate has warmed (Chen et al., 2011; Hickling et al., 2006; Mason et al., 2015; Poniatowski et al., 2020). Most multi‐species range shift studies have used a coarse spatial resolution. There is great variability between species in the extent of shift, and there is obvious conservation concern about the species that are lagging behind their climate envelope (Dawson et al., 2011). A few empirical studies explore how species' attributes can influence rates of range shift, for example their climate sensitivity, their dispersal ability and their habitat specialism (Angert et al., 2011; Fei et al., 2017; Pöyry et al., 2009; Sunday et al., 2015; Warren et al., 2001). The fact that habitat specialist species appear less able to expand into newly suitable areas (Fartmann et al., 2021; Platts et al., 2019; Warren et al., 2001) points to a key interaction between species' biology and the landscape configuration. However, the relative importance of landscape configurations in making range shifts faster in certain sub‐landscapes, and for certain species, has been predominantly explored with models (Hodgson et al., 2011; Hodgson et al., 2012; Mcinerny et al., 2007; Synes et al., 2020). Therefore, the widespread conservation advice that habitat connectivity should be enhanced to facilitate climate adaptation is based on modelling and on extrapolation from movement studies, and is biologically plausible, but lacks empirical support.

From theory and modelling it is clear that the amount and spatial pattern of habitat should affect the rate of range shifting. When there is a low coverage of breeding habitat, the total number of potential dispersers is reduced, and the distances individuals must travel in their lifetime is increased, strongly reducing the potential to colonise new regions. The main effect of habitat amount at the poleward range edge has been qualified empirically (Hill et al., 2001, 2002; Mair et al., 2014; Platts et al., 2019) but the effects of habitat spatial configuration and barriers are more subtle and difficult to quantify. One study shows that habitat “clumpiness” seems to delay climate‐driven changes in communities (Fourcade et al., 2021). There is relevant research on dispersal and inter‐population connectivity in long‐occupied landscapes (e.g., Fletcher et al., 2016; Gilbert‐Norton et al., 2010; Hartfelder et al., 2020; Resasco, 2019; Wright et al., 2020) which could be extrapolated to a multi‐generation, range‐shifting context. For example, landscape elements that are hostile may act to either retard or re‐route range expansion, and linear or stepping‐stone‐like configurations may act as conduits (Marrotte et al., 2020). Modelling has suggested that landscape “conductance” could be a useful summary metric of the speed of range shifts achievable, based on both the amount and the configuration of breeding habitat (Hodgson et al., 2012; Hodgson et al., 2016; Poniatowski et al., 2016). In brief, the breeding habitat in a landscape is converted to nodes in a network, linked by a per‐generation rate of colonisation from every node to every other; conductance between two ends of the landscape is highly correlated to the multi‐generation speed of spread of simulated species (Hodgson et al., 2012, 2016). If conductance explains a substantial proportion of the variation in range shift rates between landscapes and between species, it would provide one of the most useful and widely applicable methods for planning land conservation and restoration under climate change. That is because the impacts of losing or gaining patches of habitat in any location are very quick to quantify with conductance, and thus conservation can be spatially targeted (Hodgson et al., 2016; Williams et al., 2020).

Here, we examine both the speed and the spatial pattern of contemporary range shifts in unprecedented detail, for a taxon with diverse habitat associations. We quantify the relative contributions of landcover variables that may be beneficial or hostile and test how the habitat conductance metric competes with simple metrics of coverage and distance. Because we aim ultimately to inform conservation, we also test whether coverage of protected areas appears to enhance range shifting rates, in addition to the effects of landcover. There is some evidence that protected areas have been colonised preferentially during range expansions for a variety of taxa (Gillingham et al., 2015; Thomas et al., 2012).

We study British moths (night‐flying Lepidoptera) because they are known to be sensitive to both climate change and land‐use change (Conrad et al., 2004; Fox et al., 2014), large groups associated with different landcover types can be readily identified, and the species cover a very wide specialism‐generalism continuum (Randle et al., 2019; Waring & Townsend, 2009). We choose southerly distributed species in Great Britain whose range edge is likely to be determined by climate. We use exceptional data from a long‐running light‐trap network (Bell et al., 2020; Conrad et al., 2004) to pinpoint the time at which each species arrived at key locations outside its historic range, or, equally importantly, to indicate that a species has not yet arrived.

## 2 METHODS

### 2.1 Moth distribution data and selection of species

Fox et al. (2014) analysed the distribution trends of 673 moth species considered resident in Great Britain. They used the Frescalo technique (Hill, 2012) to control for variable survey effort over both space and time, yielding for each species a corrected estimate of its multi‐decadal distribution increase or decrease (2000–2010 vs. 1970–1999). We used their results to select 75 species that, according to Frescalo estimates, had increasing “relative reporting rate” trends (which means that they were increasing in abundance and/or range), occurred in at least 30 hectads and did not occur in northern Scotland (north of 650 km OSGB) in the period 1970–99.

We extracted all daily records for the candidate 75 species from the Rothamsted Insect Survey (RIS) database. RIS is a unique network of light‐traps distributed across Britain, recording moths every night using a standard protocol (Bell et al., 2020). After visually inspecting maps of each species' RIS records colour‐coded by year, we excluded 20 from further analysis either because their range did not appear to be expanding in any direction or because there were too few records. We also excluded from analysis one further species, Cypress Carpet, *Thera cupressata*, which was first recorded in Britain in 1988.

We extracted all available distribution data for the remaining 54 species from the National Moth Recording Scheme (NMRS) database maintained by Butterfly Conservation. This comprises species occurrence data collected by thousands of volunteer recorders, by various methods and with variable and often unknown survey effort (Fox et al., 2011). Its benefit is that it has more complete geographical coverage than the RIS survey. Therefore, we used the NMRS data to delineate the baseline geographic distribution of each species. We chose 1965–1985 as the baseline period, because this is recent enough to have many records at 1 km resolution or better, and leaves ample time to detect differential speeds of range expansion (see below). We defined the “baseline distribution” of each species for all further analyses as any 1 km^2^ that contained a record at 1 km resolution or better during 1965–1985. We considered these the most likely sources of populations that could contribute to any range expansion (and that the disadvantages of including any older records assuming that the populations were still present there would outweigh the potential advantages). A total of 91,553 records met these criteria to be included in the baseline; *c*. 19,000 were excluded for being too old, and only 3540 were excluded for having a resolution coarser than 1 km. It is unfortunately inevitable that the baseline distributions defined in this way will contain gaps due to limited survey effort, but gaps scattered through the distribution do not undermine our ability to characterise the landscape that the species most likely had to cross to reach the RIS traps (see next section).

### 2.2 Range expansion in terms of arrival at new RIS traps

The overall aim of the analysis was to determine how the speed of range expansion is determined by certain species attributes, and characteristics of the landscape being crossed. Using the RIS data, we could determine the exact date when each species was first caught at each trap location. Equally informative are traps that have been continuously running for a period of time but where a species has *not* been observed. Survival analysis offers a way to analyse these data that makes minimal assumptions about the process of range expansion (Kalbfleisch & Prentice, 2002). The dependent variable in this analysis is the time elapsed before species *s* arrives at trap *t*, or before trap *t* ceases to operate, for all valid species‐trap combinations. A species‐trap combination was considered valid only if the trap was at least 20 km from any RIS or NMRS record of the species during the baseline period of 1965–1985 (i.e., we were only interested in arrivals at locations clearly outside the species' baseline range). Thirty traps were operating continuously from 1985 to 2011, and 40 traps ceased to operate at various times in between. This means that our survival data are right‐censored—a situation that survival analyses are designed to account for. We decided to use the single first catch of each species at each trap location, rather than any more complex definition of successful colonisation. Although this does not prove there is a viable population around the trap in question, it is an important event in a range expansion scenario. It indicates that the trap is within the normal dispersal distance of a population, although what this distance is will vary with the species.

We used three classes of explanatory variables for the time until species *s* arrives (or fails to arrive) at trap *t*, allowing us to test the relative importance of habitat configuration versus other factors:

- attributes of species s, in particular habitat associations, determined from Waring and Townsend (2009)
- characteristics of the immediate surroundings of trap *t* (within 1 km)
- characteristics of the “expansion zone” in between trap *t* and the known baseline distribution of species *s* in 1965–1985, including the Euclidian distance between the trap and the three nearest baseline records, and the habitat “conductance” (see next section)

**FIGURE 1:**
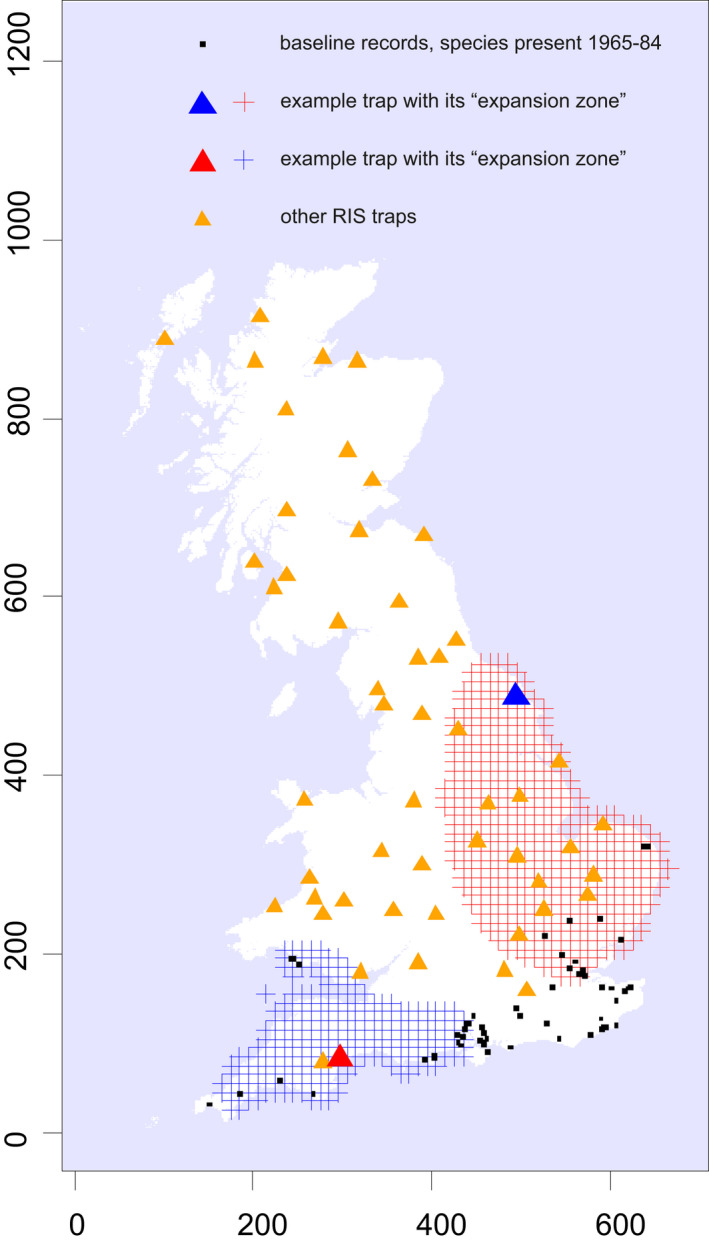
Map to illustrate the moth data sets and “expansion zones,” using an exemplar species, Webb's wainscot*, Globia sparganii*. The baseline distribution (black squares) includes records from both NMRS and RIS for 1965–84 at 1 km resolution or finer. The arrival of the species after 1984 is then analysed at all RIS traps that are >20 km from the baseline distribution (triangles). For each RIS trap, landcover and climate information is taken from a relevant “expansion zone” (shown with hatched areas for two example traps; larger triangles) to attempt to predict the time the species arrives at that trap. Spatial units are km of the British National Grid (OSGB)

The expansion zone relevant to each species‐trap combination was defined as follows. A 10 km^2^ was in the expansion zone if [the Euclidean distance from the square centre to the nearest record of species *s* plus the Euclidean distance from the square centre to trap *t*] was less than [100 km plus the minimum Euclidean distance between trap *t* and any record of species *s*]. Using this definition gives the zone a flexible shape depending on the spatial arrangement of the baseline records: some examples of the zones are given in Figure 1. We judged that this definition consistently included the regions that colonising moths would most likely pass through and that a more complex definition could not be justified by the data.

We were only interested in predicting one value for each trap: the mean expected time until colonisation. Therefore, we use the simplest possible parametric survival model (using the function *survreg* in the R package *survival*) with no change in the probability of arrival with year (exponential model). We included a frailty term to account for unpredictable differences in colonisation ability between species (similar to a random effect in a GLMM).

Two broad categorisations of our moth species gave large enough groups to fit separate effects: firstly, whether species are reportedly associated with woodland, and secondly whether species are associated with common features of anthropogenic landscapes (urban, gardens, parks, farmland, hedgerows or scrub being mentioned in the field guide; Waring & Townsend, 2009), which we henceforth termed “farmland” species (some species are both woodland‐ and farmland‐associated, i.e. two binary factors were used to classify the species).

### 2.3 Landscape and climate data

Landcover information was extracted from the CEH landcover map 2007, which is a parcel‐based interpretation of satellite data into 23 ecologically relevant broad landcover types (Morton et al., 2011). The data were supplied as a raster at 25 m resolution.

Vector maps of sites of special scientific interest (SSSIs) were obtained for England, Scotland and Wales from Natural England, Scottish Natural Heritage and Natural Resources Wales web services respectively. These were appended together using ArcGIS desktop 10.1 and converted to a raster at 25 m resolution.

Although we did not have the power to fit a reliable climate response model for each species, climate could be a major factor explaining differences in shifting rates. We hypothesised that climate variance, or the steepness of the climatic gradient, could hamper colonisation for all species no matter where their individual tolerance threshold lay. Late 20th century monthly mean temperature data for the United Kingdom were downloaded from Worldclim (worldclim.org) at 1/120 degree resolution (*c*. 1 km). Raster calculator in ArcGIS desktop 10.1 was used to calculate total growing degree days above 5°C (GDD5) over all months. GDD5 was chosen as a measure that has often been found relevant to insect development. The GDD5 raster was then converted to OSGB projection at 1 km resolution. Elevation data were also downloaded from Worldclim and converted to OSGB projection at 1 km resolution.

All subsequent analyses were performed using the *raster* package in R 3.0.1. For each expansion zone, and for a circle of radius 1 km around each RIS trap, we calculated:

- proportion coverage of broadleaved or mixed woodland
- proportion coverage of coniferous woodland
- proportion coverage of suburban land use
- proportion coverage of SSSIs
- mean GDD5
- variance of GDD5
- mean elevation
- variance of elevation

### 2.4 Conductance of woodland

We aimed to test the predictive performance of the conductance metric, proposed by Hodgson et al., 2012 to assess how landscapes could facilitate range expansion. Woodland‐associated species were most prevalent in our data set, and no other single‐habitat group had enough arrival data for separate analysis. Therefore we calculated conductance only for woodland habitat networks, following the method of Hodgson et al., 2012. Briefly, this quantifies the contribution that habitat has to reproduction and movement over multiple generations, based on its area and spatial location. The “source” for the circuit calculation included all baseline records up to 100 km further than the closest record, the “target” was the focal trap, and all woodland habitat at 1 km resolution was used to define possible routes of colonisation (more detail in Appendix A). Versions of conductance assuming different mean dispersal distances (5, 10, and 20 km), and including coniferous woodland or not, were tested.

### 2.5 Model fitting and selection

We separately fitted survival models for all species and for the subset of woodland‐associated species. The data frame used for statistical analysis is archived in Dryad (Hodgson et al., 2022). We built models by stepwise addition, considering only interactions we considered a priori to be biologically plausible. In addition, we avoided including two highly correlated variables in the same model (this applied to GDD5 variance with elevation variance; cover of deciduous woodland with cover of suburbs; and the variants of conductance with different dispersal and habitat settings). When either deciduous woodland cover or coniferous woodland cover were found to be significant predictors, we also tested whether it was more parsimonious to use combined all‐woodland cover. We considered plausible one‐way interactions between species' habitat affiliations (woodland or farmland) and one of the following variables: woodland or suburban cover within 1 km of the target trap and deciduous/coniferous woodland cover, suburban cover, elevation variance, GDD5 variance or SSSI cover within the expansion zone. After finding a putative minimum adequate model, we tried replacing variables one‐by‐one with highly correlated alternatives (the alternatives mentioned above). Where this led to a model that differed in AIC by less than 2, we included these in our results, and where relevant used model averaging based on Akaike weights to generate predictions.

## 3 RESULTS

### 3.1 Time until colonisation for all species

We first fitted a survival model including data for all 54 expanding species, comprising 2029 species‐trap combinations and 192 observed colonisations. The single most important factor explaining time until colonisation was distance between the focal trap and the nearest baseline records of the focal species (Table 1). There were also several strong effects of the landcover in the expansion zone, and of the landcover within 1 km of the trap, and some of these effects were dependent on species' habitat specialism (Table 1).

**TABLE 1:** Parameter table of one of 12 low‐AIC models to explain time until colonisation across all species. Data for all 12 models is given in Table S1; they are all similar because they involve substituting highly correlated variables. This one was chosen because it has almost the lowest AIC (delta AIC = 0.2) and illustrates the three notable interactions. Note that shorter times, and negative parameters, signify faster colonisation. Interactions are expressed as “factor:continuous variable” so that the associated tests are against the null hypothesis that the slope equals zero for the given level of the factor

Parameter	Value	Std error	*p* (z test)
Intercept	−8.600	2.145	.00006
Woodland‐associated species (Woodsp)	1.413	0.550	.010
Farmland‐associated species (Farmsp)	−6.054	2.456	.014
Distance to nearest three baseline records	1.093	0.133	<10e‐6
Proportion suburban cover within 1 km of trap	1.959	0.560	.00047
Proportion woodland cover within 1 km of trap	−0.975	0.273	.00036
Proportion broadleaf/mixed woodland cover in expansion zone (if not Woodsp)	33.020	10.124	.0011
Proportion broadleaf/mixed woodland cover in expansion zone (if Woodsp)	−5.880	5.793	>.2
Variance of GDD5 in expansion zone (if not Farmsp)	0.019	0.164	>.5
Variance of GDD5 in expansion zone (if Farmsp)	0.723	0.156	.0000035
Proportion broadleaf/mixed woodland cover in expansion zone (if Farmsp)	−25.872	9.748	.0080

When the expansion zone contained higher proportions of deciduous/mixed woodland, non‐woodland species colonised much more slowly, but this effect seemed to be cancelled out for farmland species (Table 1; −26 interaction coefficient for farmland species in addition to +33 interaction coefficient for non‐woodland species results in little effect on species that fit both categories). The proportion of deciduous/mixed woodland in the expansion zone was very highly positively correlated to the proportion of suburban landcover in the expansion zone, so models containing either of these in interaction with species' habitat affiliation had high Akaike weights (Table S1). When the expansion zone had a higher temperature (GDD5) variance or higher elevation variance, farmland species (only) colonised significantly more slowly (Table 1). GDD5 variance is so highly correlated to elevation variance that models containing either had high Akaike weights (Table S1).

**FIGURE 2:**
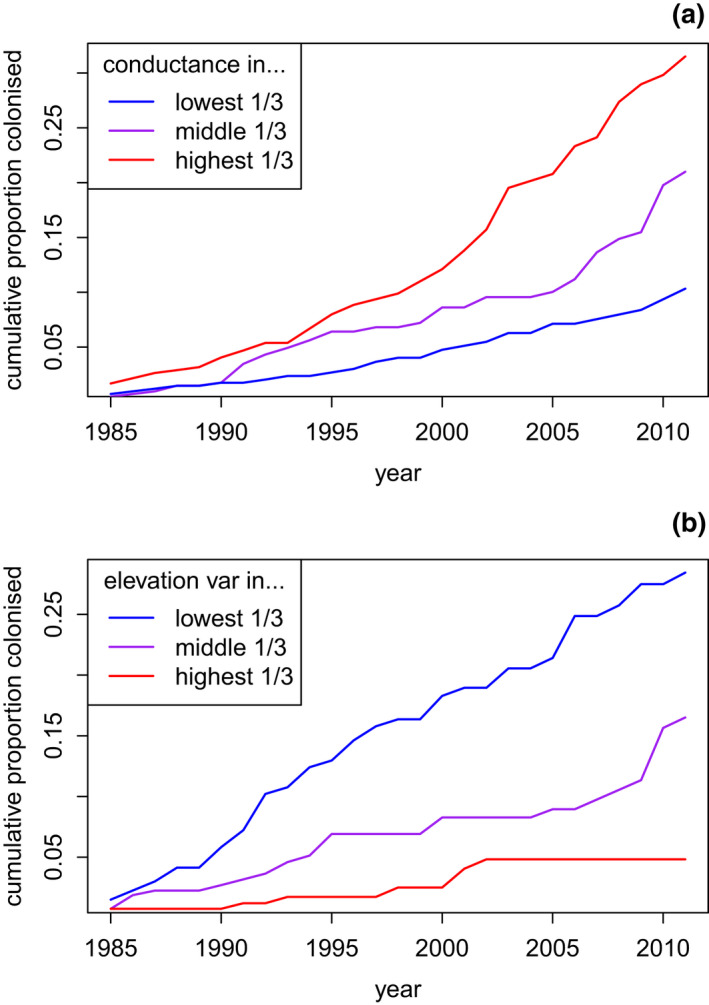
Observed “survival” curves—the cumulative proportion of target traps colonised over time—For different levels of conductance for woodland‐associated species (a), and for different levels of elevation variance for farmland‐associated species (b). The three lines represent 1/3 quantiles of the data for conductance (a) or elevation variance (b). The observed probability of colonisation at each time step is simply the number of colonisations at that time step divided by the total number of operating (non‐censored) traps at that time step

**FIGURE 3:**
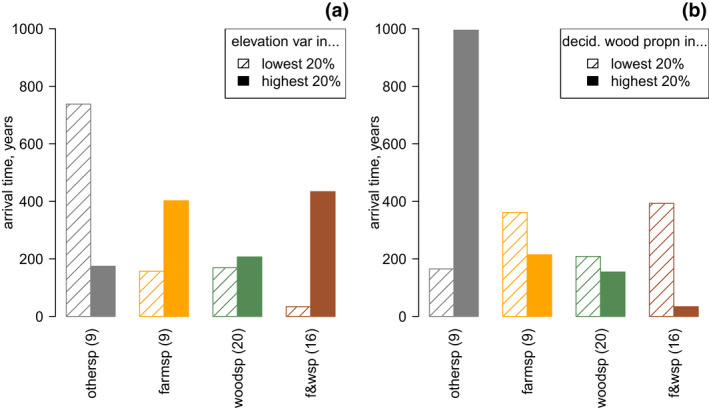
Effect sizes of realistic variation between landscapes affecting the times taken to reach 100 km beyond their baseline range, for different species types (farmland or woodland‐associated). Bar heights illustrate how geometric mean predicted arrival time changes when landscapes sit in the top 20% versus the bottom 20% of observed landscapes for either elevation variance (a) or deciduous woodland cover (b). Species are classified as farmland ‐associated, woodland‐associated, both (“f&wsp”) or neither (“othersp”), with number of species in each group given in brackets after the label. Predicted values are generated from all plausible models and averaged based on Akaike weights (see Table S1). Realistic covariation between the four variables describing the expansion zone is preserved by resampling whole rows of these variables. Distance to the nearest three baseline records is fixed at 100 km and other variables at their median observed values

The variation in landcover across Britain is large enough to cause material differences in species' ability to keep up with climate change; this can be seen in the raw data (Figure 2) and in predictions from our models for high and low levels of the observed variables (Figure 3). Predicted times to extend the range by 100 km could vary by orders of magnitude (Figure 3). In general in the average British landscape, woodland species tended to colonise faster than non‐woodland species; this can be seen in predictions from our models (Figure 3) and in a simple model including only distance and the species classifications, where woodland species colonised significantly faster (parameter = −0.571, SE = 0.176, *p*[*z*] = .0012), and the farmland classification did not have a significant effect (*p*[*z*] = .08). The relatively small group of species associated neither with farmland nor woodland exhibited some of the slowest colonisation rates overall, and rates which were strongly affected by landcover (Figure 3).

Traps with higher proportions of suburban landcover within 1 km were slower to be colonised, and traps with higher proportions of woodland within 1 km (coniferous and deciduous combined) were faster to be colonised (Table 1). No significant effects were found for the proportion coverage of SSSIs, mean GDD5 or mean elevation in the expansion zone.

### 3.2 Time until colonisation for woodland species

We refined our model looking only at the subset of 36 species associated with woodland habitats, comprising 1250 species‐trap combinations and 139 observed colonisations. Here, we introduced the new explanatory variable of woodland conductance across the expansion zone. Where broadleaved/mixed woodland conductance was higher, colonisation was significantly faster (*p* = .00001, Table 2). The measures of woodland proportional cover in the expansion zone and within 1 km of the focal trap were no longer significant in this model, indicating that the conductance measure successfully captured the functional effect of habitat availability, and improved on it by accounting for its spatial arrangement as well. However, distance between the trap and the baseline distribution was still a highly significant variable (both distance and conductance improved the model although they are correlated) (Table 2). The detrimental effect of elevation or climatic (GDD5) variance on farmland species was still evident as in the full model (Table 2, Table S2). Traps with higher proportions of suburban landcover within 1 km were slower to be colonised, as in the full model.

**TABLE 2:** Parameter table of the lowest‐AIC model to explain time until colonisation for woodland associated species. Note that shorter times, and negative parameters, signify faster colonisation. Interactions are expressed as “factor:continuous variable” so that the associated tests are against the null hypothesis that the slope equals zero for the given level of the factor. Lower‐AIC models are detailed in Tables S2 and S3

Parameter	Value	Std error	*p*(*z* test)
Intercept	−5.54	1.61	.00057
Farmland‐associated species (Farmsp)	−7.57	1.58	<10^−5^
Proportion suburban cover within 1 km of trap	2.38	0.62	.00013
Distance to nearest three baseline records	1.06	0.14	<10^−6^
Conductance of woodland across expansion zone	−0.44	0.10	.00001
Variance of elevation in expansion zone (if not Farmsp)	−0.07	0.13	>.5
Variance of elevation in expansion zone (if Farmsp)	0.69	0.14	<10^−6^

The most parsimonious conductance measure assumed a mean dispersal distance of 5 km per generation and treated only deciduous/mixed woodland as habitat. However, any of our conductance measures (including coniferous woodland as habitat; assuming different dispersal distances) were highly significant (*p* < .001) if substituted for the former (Table S3).

## 4 DISCUSSION

We present strong evidence that variation in range‐shifting rates both between landscapes and between species can be explained by aspects of habitat availability and configuration. The design of this study—using specific monitored arrival locations and years—gives a more nuanced picture than any previous multi‐species studies of climate‐induced range shifting. The most novel result is to show that conductance is a valuable metric to explain range expansion success: conductance measured on woodland habitat predicts the range expansion rates of woodland species, even when the metric is not tailored to the dispersal capabilities or exact life history requirements of each species. It is clearly more informative than the proportion of woodland in the landscape. This is an important empirical test of the theory in Hodgson et al. (2012), and provides some confidence that the metric could be used for conservation planning, to ascertain how to make landscapes more permeable.

If unfavourable landscape configurations and hostile habitats are restricting species' capacity to adapt to climate change, this has serious conservation implications. The risks are clear from modelling and from knowledge of biological mechanisms, but there is only limited empirical evidence to date; for example, showing that community change is slower in sites surrounded by intensive land use (Oliver et al., 2017) or with more ‘clumped’ spatial configuration (Fourcade et al., 2021). We have shown that, within a set of species classified as expanding, that woodland seems to hamper the range expansion of non‐woodland species, (Table 1). This is concordant with some small‐scale dispersal studies showing that insects of open habitats avoid entering woodland (e.g., Öckinger & Smith, 2008), but it is perhaps surprising that the effect is seen so strongly in the British landscape where woodland cover is generally low (*c*. 10%), and large, continuous woodland blocks uncommon. We also found that regions of high elevation variance or climatic variance seem to restrict the expansions of “farmland” species (those associated with anthropogenic habitats; Tables 1 and 2).

It has previously been reported (for butterflies, Warren et al., 2001 and other invertebrates, Platts et al., 2019) that farmland species are more likely to be keeping pace with climate change. It seems reasonable to assume that the landscape is relatively permeable for them as their habitat is very common (in Western Europe at least) and there has not previously been any evidence that any specific landscape factors could hamper their range expansion. There are several plausible mechanisms that could explain the effects of high elevation variance or climatic variance on them. First, there could be an effect of temperature (GDD5) per se: where there is high temperature variance, species will encounter temperatures that contrast more strongly with their baseline distribution. For a given global warming rate and all else being equal, isotherms will move at a lower rate of km/year in an area with higher climatic variance. Alternatively, it could be that hills present a physical barrier to dispersal, or that upland areas contain less habitat for farmland species (perhaps, fewer hedgerows or floral resources for example). Because these variables are all very strongly correlated it is not feasible to distinguish them with the data we have. Nevertheless, it is novel to pinpoint any of the factors causing slowing of range shifts, and these results have important implications for managing the adaptation of biodiversity to climate change, whether or not managers can influence the barriers themselves.

There is much interest in whether conservation actions can facilitate species range shifts, and some evidence that protected areas are disproportionately used by species that are undergoing range shifts (Gillingham et al., 2015; Thomas et al., 2012). However, we found no evidence that the availability of SSSIs affected the arrival times of our species. In fact, SSSI cover seemed to impede range expansion by farmland species, but this effect dropped out when the stronger effect of elevation/climate variance was included in the model. As in many parts of the world, protected area coverage is biased towards high‐elevation areas in Britain. We believe this bias may be masking any small positive effect of conservation management on our focal moth populations.

The landcover within 1 km of each trap affected the probability of moths colonising, and seemed to do so regardless of species' specialism. The positive effect of both coniferous and broadleaved woodland, and the negative effect of suburban areas suggests to us that these effects may be due to the amount of competing light which can reduce the efficiency of moth traps. It has been reported before that traps are more effective in woodland and less effective in urban areas (Bowden, 1982). It is also plausible that landcover close to the trap affects the numbers of different moths in the area and their chance of coming close enough to perceive the light (e.g., Boyes et al., 2021).

### 4.1 Conductance as a predictor of range expansion rate

We show for the first time that landscapes with higher conductance values (metric described in Hodgson et al., 2012, 2016) are crossed more rapidly by real species. We measured the conductance of woodland networks and tested its effect on woodland‐associated species, because this gave us the largest category of similar species. The conductance metric is potentially very useful for conservation planning under climate change because it can rapidly (a) show which landscapes are more permeable than others; (b) pinpoint the most crucial “stepping stones” likely to be used on the way to the range expansion target; and (c) pinpoint “bottlenecks” where habitat re‐creation would have maximal impact on range‐shifting (Hodgson et al., 2012, 2016; Travers et al., 2021; Williams et al., 2020). Conductance is based on biological mechanisms of reproduction and dispersal, which are then simplified to rates of arrival from any habitat‐containing cell to any other. Its results are clearly sensitive to variables we do not know with certainty for many species: exactly which habitats support breeding; where populations originally occurred (the “source” for range expansion), and mean dispersal distance. Therefore, it is invaluable to test its predictive performance in scenarios of realistic data availability. Our 35 woodland moth species are not all strict woodland specialists, their “baseline” distributions will be subject to some recording gaps and their individual dispersal distances were not known. Despite all these factors, the estimated conductance was a very highly significant predictor of their time of arrival at “target” trap locations. Our sensitivity analysis assuming different dispersal distances and different classes of woodland showed that all versions of conductance were useful. The conservation implications of this are very promising: if landscape configuration can be managed to increase conductance (even if that conductance is measured using imprecise parameters), there is hope that this will improve the functional permeability of that landscape to many species.

When considering the applicability of these findings for conservation, note that our analysis excluded species whose cool range margins were receding or static. This group of excluded species could include those experiencing very low habitat connectivity or restricted dispersal ability, so an improvement in landscape configuration may help them in a similar way to our included species. However, the group is also likely to include species where other complicating factors are influencing their range margin (factors that have been found in other studies, e.g., Mason et al., 2015, Platts et al., 2019). To mention a few: species may be declining in response to threats unrelated to climate change; the climate variables that have strong trends (e.g. spring temperature) may not be the ones with a large effect on some populations; or biotic factors may limit the range edge. Therefore, maintaining and improving connectivity is only one of a range of conservation policies needed under climate change (Oliver et al., 2012).

## 5 CONCLUSIONS

We have elucidated how species' attributes interact with landscape characteristics to create highly heterogeneous patterns of shifting at cool range margins. We have shown that contemporary range shifts are being influenced by habitat configuration—going beyond previous studies focussing on the coverage of habitat. Furthermore, we have demonstrated the predictive strength of the conductance metric that integrates habitat amount and configuration between specific source and target locations. In showing that some landscape factors hamper range expansion for certain groups, (as opposed to the simple effect that breeding habitat speeds expansion), we challenge the assumption that relatively mobile species, using widespread habitats, face no geographical barriers to range expansion. This is especially noteworthy since we only included species that showed some degree of expansion from their baseline distribution. Conductance, and other predictors of range shifts, can provide a foundation for developing coherent conservation strategies to manage range shifts for entire communities.

## CONFLICT OF INTEREST

The authors declare no conflicts of interest.

## AUTHOR CONTRIBUTIONS

JAH conceived and planned the study; performed analyses; interpreted results and wrote the paper. ZR, CRS, and THO advised on selection of study species; pre‐processed and provided data; interpreted results and edited the paper.

## Supporting information


**Appendix A** Conductance method

## Data Availability

The derived data that support the survival models in this study are openly available in Dryad (https://doi.org/10.5061/dryad.dr7sqvb1k). The original data were obtained from several third parties as follows, and can be accessed by other researchers. Rothamsted Insect Survey data are available from Rothamsted Research. Restrictions apply to the availability of these data, which were used under license for this study. Data are available at https://insectsurvey.com with the permission of Rothamsted Insect Survey. National Moth Recording Scheme data are available from Butterfly Conservation. Restrictions apply to the availability of these data, which were used under license for this study. Data are available at www.butterfly‐conservation.org with the permission of Butterfly Conservation. The CEH Land Cover Map is available from UKCEH. For this study, the data were used under a license for academic research. Version 1.2 of the data are now available for non‐commercial use at the NERC Environmental Information Data Centre (https://doi.org/10.5285/a1f88807‐4826‐44bc‐994d‐a902da5119c2; https://catalogue.ceh.ac.uk/documents/c0078881‐7d5a‐4641‐91e2‐c271426bc8a1). Temperature and elevation data are available in the public domain from www.worldclim.org. Sites of Special Scientific Interest spatial layers are available under an Open Government License from Natural England (https://naturalengland‐defra.opendata.arcgis.com/datasets/Defra::sites‐of‐special‐scientific‐interest‐england/about), Natural Resources Wales (catalogue permalink https://libcat.naturalresources.wales/folio/?oid=98776, data download link http://lle.gov.wales/catalogue/item/ProtectedSitesSitesOfSpecialScientificInterest/?lang=en) and NatureScot (previously Scottish Natural Heritage) (https://spatialdata.gov.scot/geonetwork/srv/eng/catalog.search;jsessionid=B2CAEB976C98B0DF0D8B69D26755F34B#/metadata/ECA527A8‐DC9A‐49F3‐8911‐F4CF9C3019A5).
