# Supplementary material for: Where and why are species' range shifts hampered by unsuitable landscapes?
Source: Glob Chang Biol. 2022 May 19;28(16):4765–74. doi: 10.1111/gcb.16220 (PMC9540991; doi:10.1111/gcb.16220)
Supplement: Supplementary file 1 — Appendix A Conductance method [file GCB-28-4765-s001.docx]

# Supplementary material for: Where and why are species’ range shifts hampered by unsuitable landscapes?

Jenny A Hodgson, Zoë Randle, Chris R Shortall, Tom H Oliver

## Appendix A: Conductance method

Conductance (Hodgson et al 2012; Hodgson et al 2016) quantifies the contribution that habitat makes to reproduction and movement over multiple generations, based on its area and spatial location. It predicts how quickly a species starting at the "source" location could colonise (first successful arrival at) the “target” location. The source for the circuit calculations here included all baseline records up to 100 km further than the closest record, the target was the focal trap, and all woodland habitat at 1 km resolution was used to define the intervening network. Versions of conductance assuming different mean dispersal distances (5, 10 and 20 km), and including coniferous woodland or not, were tested.

Circuit conductance (Hodgson et al 2012) between a source and a target, across a landscape with $N$ intermediate cells containing habitat, is calculated by $K=V\times c_{\mathrm{target}}$, where $V=\mathbf{M}^{-1}c_{\mathrm{source}}$. $\mathbf{M}$ is a $N \times N$ matrix with elements $M_{\mathrm{ij}}= \delta_{\mathrm{ij}}\sum_{l} c_{\mathrm{jl}}-c_{\mathrm{ij}}$, where $c$ is the single step conductance (rate of colonisation) between any two cells, $i$ and $j$ index the $N$ intermediate cells, $l$ indexes the $N$ cells together with the source and target, and $\delta_{\mathrm{ij}}=\left\{ \begin{aligned} 1, & i =j \\ 0, & i \neq j \end{aligned} \right.$. $c_{\mathrm{source}}$ and $c_{\mathrm{target}}$ are vectors of length $N$ of conductance values between each intermediate cell and the source or target. Should there be multiple targets or source cells the contributions from each are simply added together to produce each $c$. The rates of colonisation between one cell and another could be calculated using any kind of dispersal assumptions (as long as they are symmetrical (*c_ij_* = *c_ji_*). Here, we use a simple negative exponential dispersal kernel, and assume that both emigration rate and immigration rate are proportional to the fractional coverage of habitat (in this case, woodland) in the landscape cells (in this case, 1 x 1 km cells). Additionally, to speed up our conductance calculations, we simplified the circuit so that each intermediate circuit node was a 10 x 10 km cell, but precise rates of colonisation were determined at the 1 x 1 km resolution and then summed to produce the *c_ij_*_._

## Supplementary Tables

#### Table S1 – Akaike weights for 12 competing models explaining the time until colonisation with all moth species included. These 12 all included the significant main effects noted in table 1 (Woodland-associated species (Woodsp); Farmland-associated species (Farmsp); Distance to nearest 3 baseline records; Proportion suburban cover within 1 km of trap; Proportion woodland cover within 1 km of trap). They differed by the inclusion of interactions, and the substitution of certain correlated variables in interactions, as enumerated in the first three columns. The model shown in Table 1 is marked by a * by the dAIC.

| Climate variable in farm spp interaction | Landcover variable in farm spp interaction | Is wood zone: woodland spp interaction included? | AIC | dAIC | Akaike weight |
| --- | --- | --- | --- | --- | --- |
| elev | wood | N | 2124.7 | 0.42 | 0.121 |
| elev | wood | Y | 2124.8 | 0.45 | 0.119 |
| elev | suburb | N | 2124.7 | 0.44 | 0.120 |
| elev | suburb | Y | 2126.2 | 1.86 | 0.059 |
| elev | neither | N | 2124.3 | 0.00 | 0.149 |
| elev | neither | Y | 2126.0 | 1.73 | 0.063 |
| GDD5 | wood | N | 2126.0 | 1.72 | 0.063 |
| GDD5 | wood | Y | 2124.5 | 0.21* | 0.134 |
| GDD5 | suburb | N | 2125.4 | 1.06 | 0.088 |
| GDD5 | suburb | Y | 2126.5 | 2.20 | 0.050 |
| GDD5 | neither | N | 2128.1 | 3.80 | 0.022 |
| GDD5 | neither | Y | 2129.1 | 4.74 | 0.014 |

#### Table S2 – parameter table for second-best model for woodland species (delta-AIC 0.73), using GDD5 variance instead of elevation variance

| **Parameter** | **Value** | **Std error** | **P (z test)** |
| --- | --- | --- | --- |
| Intercept | -5.63 | 1.89 | 0.00298 |
| Farmland-associated species (Farmsp) | -10.3 | 2.34 | 0.00001 |
| Proportion suburban cover within 1 km of trap | 2.35 | 0.62 | 0.00015 |
| Distance to nearest 3 baseline records | 1.06 | 0.14 | <10^-6 |
| Conductance of woodland across expansion zone | -0.433 | 0.10 | 0.00001 |
| Variance of GDD5 in expansion zone (if not Farmsp) | -0.0571 | 0.18 | >0.5 |
| Variance of GDD5 in expansion zone (if Farmsp) | 0.972 | 0.23 | 0.00003 |

#### Table S3 – model comparison for woodland species where conductance is calculated in different ways.

*bm = broadleaved/mixed

| conductance using dispersal distance (km) | conductance using habitat | climate: farmland species interaction with | AIC | dAIC | significance of conductance variable in model (p, chi-sq test) |
| --- | --- | --- | --- | --- | --- |
| 5 | bm* woodland | elevation | 1471.7 | 0 | 0.000002 |
| 5 | bm woodland | GDD5 | 1472.5 | 0.8 | 0.000003 |
| 5 | all woodland | elevation | 1474.5 | 2.8 | 0.000009 |
| 5 | all woodland | GDD5 | 1475.1 | 3.4 | 0.000011 |
| 10 | bm woodland | elevation | 1476.7 | 5 | 0.000027 |
| 10 | bm woodland | GDD5 | 1477.3 | 5.6 | 0.000035 |
| 10 | all woodland | elevation | 1478.5 | 6.8 | 0.000073 |
| 10 | all woodland | GDD5 | 1479.2 | 7.5 | 0.000098 |
| 20 | bm woodland | elevation | 1482.2 | 10.5 | 0.000492 |
| 20 | bm woodland | GDD5 | 1482.4 | 10.7 | 0.000519 |
| 20 | all woodland | elevation | 1482.7 | 11 | 0.000663 |
| 20 | all woodland | GDD5 | 1483.7 | 12 | 0.001091 |
